# Phylogeography of Saproxylic and Forest Floor Invertebrates from Tallaganda, South-eastern Australia

**DOI:** 10.3390/insects3010270

**Published:** 2012-02-29

**Authors:** Ryan C. Garrick, David M. Rowell, Paul Sunnucks

**Affiliations:** 1 Department of Ecology and Evolutionary Biology, Yale University, New Haven, CT 06520, USA; 2 Present address: Department of Biology, University of Mississippi, University, MS 38677, USA; 3 Department of Evolution, Ecology and Genetics, Research School of Biology, Australian National University, Canberra, ACT 0200, Australia; E-Mail: David.Rowell@anu.edu.au; 4 Australian Centre for Biodiversity, School of Biological Sciences, Monash University, Clayton, VIC 3800, Australia; E-Mail: paul.sunnucks@monash.edu

**Keywords:** biodiversity, dead wood, endemism, montane refuges, population genetics

## Abstract

The interaction between physiogeographic landscape context and certain life history characteristics, particularly dispersal ability, can generate predictable outcomes for how species responded to Pleistocene (and earlier) climatic changes. Furthermore, the extent to which impacts of past landscape-level changes ‘scale-up’ to whole communities has begun to be addressed via comparative phylogeographic analyses of co-distributed species. Here we present an overview of a body of research on flightless low-mobility forest invertebrates, focusing on two springtails and two terrestrial flatworms, from Tallaganda on the Great Dividing Range of south-eastern Australia. These species are distantly-related, and represent contrasting trophic levels (*i.e.*, slime-mold-grazers *vs.* higher-level predators). However, they share an association with the dead wood (saproxylic) habitat. Spatial patterns of intraspecific genetic diversity partly conform to topography-based divisions that circumscribe five ‘microgeographic regions’ at Tallaganda. In synthesizing population processes and past events that generated contemporary spatial patterns of genetic diversity in these forest floor invertebrates, we highlight cases of phylogeographic congruence, pseudo-congruence, and incongruence. Finally, we propose conservation-oriented recommendations for the prioritisation of areas for protection.

## 1. Introduction

### 1.1. Understanding the Past to Predict and Manage for the Future

With a basic understanding of species’ biology and landscape history, the analysis of present-day spatial patterns of genetic diversity can yield insights into past evolutionary processes [1]. Phylogeographic studies explicitly consider how biogeographic landscape context has contributed to such patterns, and thus provide a springboard for subsequent studies that attempt to tease apart the relative importance of selection (local adaptation, a directional process) versus genetic drift (stochastic lineage sorting, a random processes) in driving geographic patterns of differentiation, and ultimately, speciation [2]. This line of research has direct relevance to conservation biology, given considerable debate surrounding the importance of conserving adaptive genetic variation versus lineages with distinct evolutionary histories as reflected by neutral genetic variation [3,4,5]. Similarly, given that invertebrate phylogeographic studies frequently uncover morphologically cryptic species complexes and extremely fine-scale local endemism, this work has shown that conservation strategies focused at or above the species-level, or formulated on the basis of more mobile vertebrates, are likely to be inadequate [6]. Such insights highlight the advantages of affording protection to biogeographic areas that harbour many irreplaceable evolutionary lineages, rather than focusing solely on formally recognised species [4]. Another important application of phylogeography lies in better understanding how populations, species, and species assemblages have responded to past climatic changes, including Pleistocene glaciations [7]. These studies have the potential to provide critical insights into how species might be affected by, and respond to, future environmental change such as global warming [8,9,10,11].

### 1.2. Scaling-up from Single Species to Whole Communities

Geographic areas that retained multiple refuges and were not extensively glaciated during Pleistocene climatic cycles are excellent natural laboratories for studying impacts of past climate change on regional biota. This is because each refuge area represents a semi-independent replicate from which phylogeographic inferences can be drawn, thereby permitting a greater appreciation of inherent variability in species’ responses [12]. Substantial additional power for historical inference from such regions is gained by considering multiple co-distributed species [13]. A major strength of this comparative phylogeographic approach lies in its ability to distinguish species-specific (idiosyncratic) responses from landscape-level process [14,15]. Ultimately, testing hypotheses that apply to all members of an ecosystem or community provides a framework for assessing the relative influence of evolutionary processes shaping regional biotas over broad temporal and spatial scales [13]. Scaling-up from individual species to whole communities can be challenging, but considerable analytical advances have been made in recent years [13,16,17,18,19,20]. Furthermore, some generalities are beginning to emerge from syntheses of phylogeographic inferences for diverse sets of co-distributed invertebrates [21,22,23,24,25,26,27,28,29]. For example, in landscapes that remained mostly free of ice sheet advances during historical climatic cycles, montane invertebrates often show considerable population-level genetic differentiation, as sheltered habitat refuges repeatedly served as reservoirs of biodiversity throughout the Pleistocene [30]. However, given that interactions among landscape setting, palaeoclimatic history, and organismal biology can be complex, we caution against generalisation. In this context, the underrepresentation of certain regions and taxa in phylogeographic studies (e.g., Southern Hemisphere terrestrial invertebrates [31]) is concerning.

### 1.3. Saproxylic and Forest Floor Invertebrates as Models for Phylogeography

Limited dispersal ability facilitates historical inference [32,33], and the genetic signatures of past range expansion, contraction and population divergence seen in the genomes of flight-limited or flightless invertebrates have been very informative about the number and locations of ancient refugia [30]. Furthermore, fine-scale patterns of population differentiation have revealed that diversity in low mobility invertebrates may predict local biodiversity hotspots in co-distributed vertebrates [34,35]. Owing to their sedentary nature, saproxylic invertebrates (*i.e.*, those that depend on dead wood microhabitats such as rotting logs) are exceptionally useful for detecting fine-scale geographic patterning resulting from long-acting processes such as climatic cycles, because viable populations can persist in isolated refugia too small to support vertebrates [34,36,37,38]. Indeed, owing to their ecological specialization, saproxylic invertebrates are likely to have closely tracked the changing distribution of moist forests throughout the Pleistocene and earlier. Even over ecological timescales, rotting logs are among the most stable naturally-occurring above-ground terrestrial habitats. Large-diameter decomposing logs on the forest floor may be habitable for over 70 years on the ground [39], and log interiors can remain very moist throughout the year and are buffered from extremes in temperature fluctuation. Many invertebrates are surprisingly long-lived (e.g., 15–30 year old trapdoor spiders, [40]). Even for short-lived sedentary species, however, the longevity of rotting logs provides the potential for multiple organismal generations within a single log [41], which favours the retention of phylogeographic signal. Moreover, saproxylic invertebrate assemblages are important in their own right. For example, they play a critical role in the decomposition of dead wood and contribute to nutrient cycling and soil formation [42], encompass a significant proportion of total biodiversity in forest ecosystems [43], and often include many naturally rare species [44].

### 1.4. Landscape Context and Palaeoclimatic History of Tallaganda, South-Eastern Australia

In contrast to the boreal landscapes of Europe and North America [45], Australia remained almost entirely free of ice sheet advances during Pleistocene glacial periods: glaciations of mainland Australia were restricted to a small, high-altitude region of the Snowy Mountains (the Kosciuszko Massif, 2228 metres at the peak [46]). Accordingly, genetic architectures of temperate south-east Australian taxa might have been shaped by different processes to those that impacted Northern Hemisphere biota. For example, the prominent role of extensive range expansions out of southern Pleistocene refugia, and recent recolonisation of northern Europe via common dispersal routes following retreat of Quaternary ice sheets [45,47,48], may not be reflected in Australian regional biotas. Instead, species from non-glaciated temperate Southern Hemisphere biogeographic landscapes may have a long history of co-association, coevolving *in situ* over long periods within local, stable refuges that repeatedly served as centres for the retention of biodiversity. Some support for the latter scenario is provided by biogeographic and phylogeographic studies of moist-forest-dependent amphibians, reptiles and invertebrates from the Great Dividing Range of south-eastern Australia [49,50,51,52,53,54,55]. However, detailed comparative phylogeographic studies centred in temperate montane regions of Australia are few.

Tallaganda, comprising Tallaganda State Forest and National Park, Badja State Forest, and parts of Gourock and Deua National Parks, lies on the Gourock Range—a physiographically isolated section of the Great Dividing Range. This *c*. 100 km (north-south) by 3–17 km (east-west) forested region is bounded to the east and west by the ancient Shoalhaven and Murrumbidgee valleys, and to the north by the Lake George basin, all of which supported grassland rather than forest throughout the Pleistocene and to the present [56,57]. During the cool and dry periglacial episodes that repeatedly affected south-eastern Australia throughout the *c*. 19 glacial-interglacial cycles of the Pleistocene [58,59], moist forest habitats repeatedly contracted into lower-lying, sheltered gullies in topographically heterogeneous montane landscapes, to be replaced at higher altitudes by alpine grassland. During interglacial periods, forest habitats expanded, forming continuous forest in the southern ranges [56].

Because the Great Dividing Range has a long history of geological stability (≥50–70 million years, MY), land forms have been preserved [59]. This topography would have exerted a strong influence on historical distributions of moist forest vegetation at Tallaganda by promoting local orographic rainfall [57]. While eucalypt forests are at present continuously distributed across Tallaganda, during periglacials, there was likely to have been an abrupt vegetation boundary marking the transition from the forested Gourock Range to the surrounding flat, treeless tablelands [57]. Accordingly, most of Tallaganda has been ecologically isolated from surrounding areas during the Pleistocene. These landscape characteristics, together with the very high density of rotting logs on the forest floor [39], make Tallaganda an excellent setting for studying fine-scale evolutionary responses to past climatic cycles of saproxylic and forest-dependent invertebrates.

## 2. Approach

### 2.1. Prior Expectations

Two overarching questions have been pertinent throughout the invertebrate comparative phylogeography research program at Tallaganda: to what extent does topography, particularly drainage networks, predict spatial-genetic patterns within species, and did historical climatic cycles promote the similar responses among distantly-related species? These broader questions are being addressed via the following set of four spatially and/or temporally explicit predictions that are applicable to, and testable in, all members of the forest floor community at Tallaganda.

**Figure 1:**
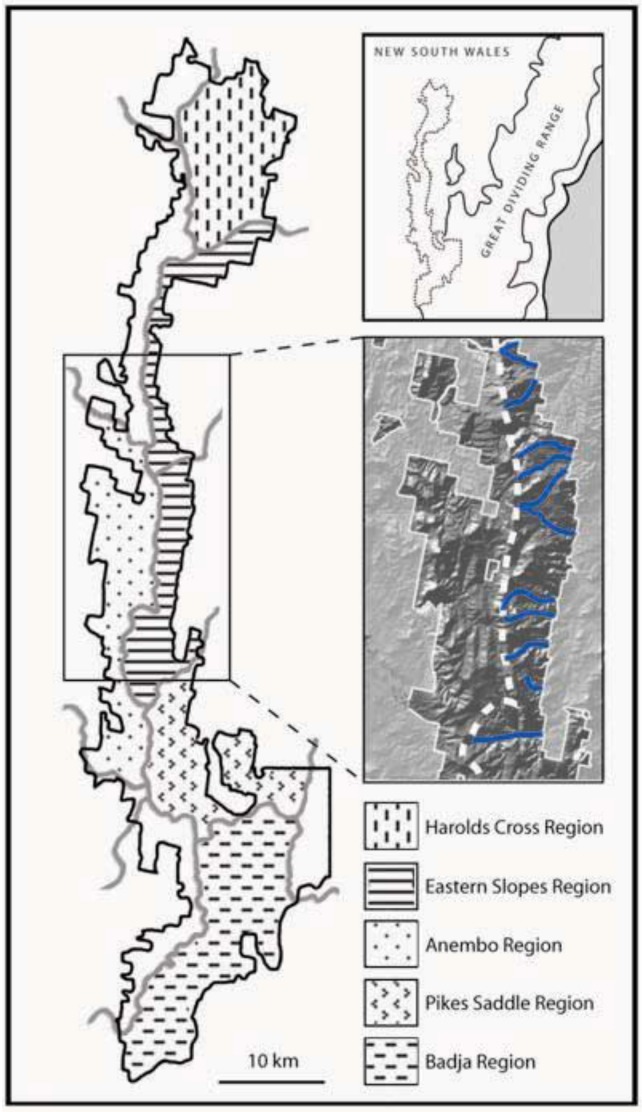
Five *a priori* microgeographic regions of Tallaganda described by Garrick *et al*. [60]. Major and minor catchment divisions are represented by thick gray lines, with the study area circumscribed by black lines. Regional affiliations of areas in white were not delineated in the original landscape model. Upper inset: physiogeographic context of Tallaganda. Lower inset: Relief map of part of the Eastern Slopes Region. Given its easterly aspect, this region is (and no doubt was historically) exposed to high levels of orographic rainfall, in contrast to west-facing slopes. Thus the numerous east-west oriented gullies and creeks (blue lines) potentially served as moist, low-lying Pleistocene refuges for saproxylic invertebrates. The main ridgeline is marked by the dashed white line.

- *Prediction 1*: Given the long-term stability of Tallaganda’s topography, five *a priori* regions delineated partly on the basis of drainage divisions will harbour distinct gene pools of low mobility forest floor invertebrates (see Figure 1 for geographic boundaries and nomenclature). These gene pools may be evident either as monophyletic haplotype clades on an estimated mitochondrial DNA (mtDNA) gene tree, or when diploid nuclear genetic markers are available, as natural genotypic clusters.
- *Prediction 2*: Based on topographic and hydrological characteristics of Tallaganda, the Eastern Slopes Region is predicted to have contained the highest concentration of suitable refuges for wet-adapted forest floor invertebrates during periglacial periods (Figure 1, lower inset). Thus, within Eastern Slopes Region we expect high genetic diversity, deep molecular divergences among sampled haplotypes, and early-branching DNA sequences or lineages on an outgroup-rooted gene tree to be concentrated here.
- *Prediction 3*: Owing to the repetitive nature of historical climatic cycles, their impacts will be detectable on multiple timescales. When inferences are based on DNA sequence data, molecular dating approaches should reveal two or more mostly non-overlapping timescales of lineage divergence and/or demographic changes.
- *Prediction 4*: The strongest evidence for postglacial range expansion is expected within catchments that experienced the greatest retraction of moist forests during Pleistocene periglaciation. Harolds Cross Region, Anembo Region, Pikes Saddle Region, and Badja Region are predicted to have been dominated by dry woodland or treeless steppe during cool dry periods. Accordingly, these regions are expected to show gene tree topologies that are indicative of recent population growth and geographic expansion, and reduced genetic diversity compared to Eastern Slopes Region, the area of relative stability and/or putative geographic source of recolonists.

### 2.2. Focal Invertebrates

In the present paper we focus on springtails and flatworms, which are closely tied to the saproxylic habitats, and have been the subject of several detailed phylogeographic analyses at Tallaganda. Springtails and flatworms also have sufficiently long histories at Tallaganda to yield well-resolved mtDNA gene tree topologies, and to facilitate reconstructions of past demographic changes.

**Figure 2:**
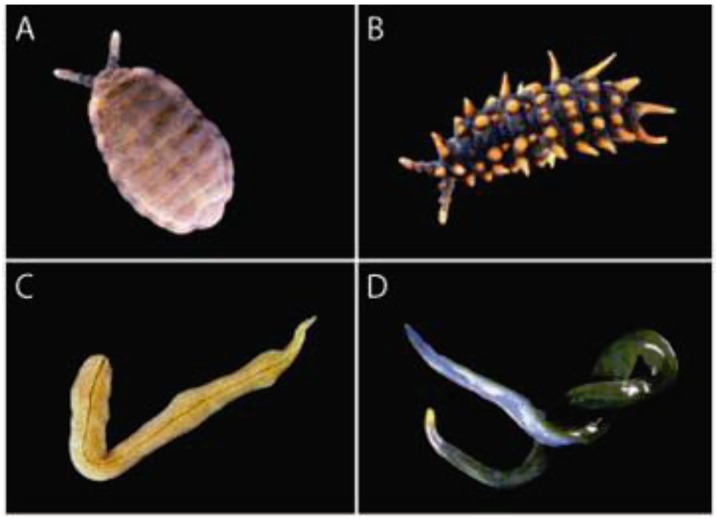
Focal invertebrate species include two springtails (Collembola, top) and two terrestrial flatworms (Platyhelminthes, bottom). All are associated with dead wood on the forest floor at Tallaganda, south-eastern Australia. (**A**) Pseudachorutinae sp. (new genus and species). (**B**) *Acanthanura* sp. (new species). (**C**) *Artioposthia lucasi* [65]. (**D**) *Caenoplana coerulea* [66].

*Springtails*: Although as-yet undescribed, the two species of saproxylic Collembola (family Neanuridae) have been clearly characterised. One represents a new genus and species in the subfamily Pseudachorutinae, and the other is a new species of *Acanthanura* in the subfamily Uchidanurinae (Figure 2A and B, respectively; Pseudachorutinae sp. and *Acanthanura* sp. herein). Both are unusually large (often >5 mm long), dorsoventrally-flattened, and graze on plasmodial slime moulds found inside moist, well-decomposed, large-diameter rotting logs [61]. They are soft-bodied, and thus extremely susceptible to desiccation. Accordingly, dispersal is thought to be very limited.

*Flatworms*: The two species of flatworm, *Artioposthia lucasi* and *Caenoplana coerulea* (suborder Terricola; Figure 2C and D, respectively) belong to genera believed to be Gondwanan relicts [62,63]. Both are hermaphrodites, prone to desiccation, sensitive to sunlight and heat, and reliant on invertebrate prey [62,63,64]. While adults are found both in and under rotting logs, their tanned egg cocoons are usually found within moist logs. *Caenoplana coerulea* has a ciliated creeping sole, which is associated with mobility. Conversely, *Artioposthia lucasi* has no creeping sole and excretes a sticky mucus layer, features associated with lower mobility, and on average, this species is usually about half the weight of *Caenoplana coerulea*. Preliminary data from desiccation experiments suggest that the capacity for persistence in and dispersal through moderately dry microhabitats is greater in *Caenoplana coerulea*.

## 3. Spatial Patterns of Intraspecific Diversity

### 3.1. Geographically Localized Genetic Lineages

**Figure 3:**
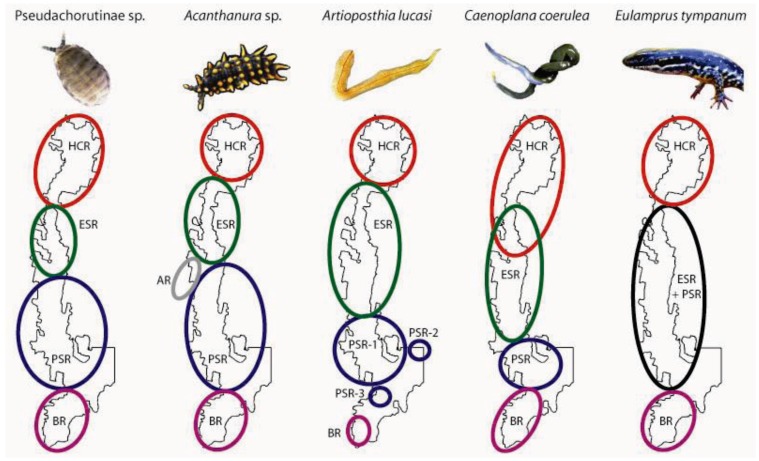
Approximate spatial distribution of genetic populations of two springtails, two terrestrial flatworms, and a co-distributed terrestrial water skink at Tallaganda. Genetic populations were delineated using nuclear genotypes plus mtDNA (springtails), or mtDNA sequences only (remaining taxa). Labels reflect the *a priori* microgeographic region(s) to which a given population is most strongly associated (see Figure 1).

Very fine-scale local endemism, as reflected by marked population structure over distances on the order of tens of kilometres or less, appears to be common in low-mobility saproxylic invertebrates [34,67,68,69,70,71,72,73]. At Tallaganda, several taxa exhibit abrupt transitions between deeply divergent genetic lineages (Figure 3). Of these, the Pikes Saddle / Badja Region break is the most taxonomically pervasive.

**Figure 4:**
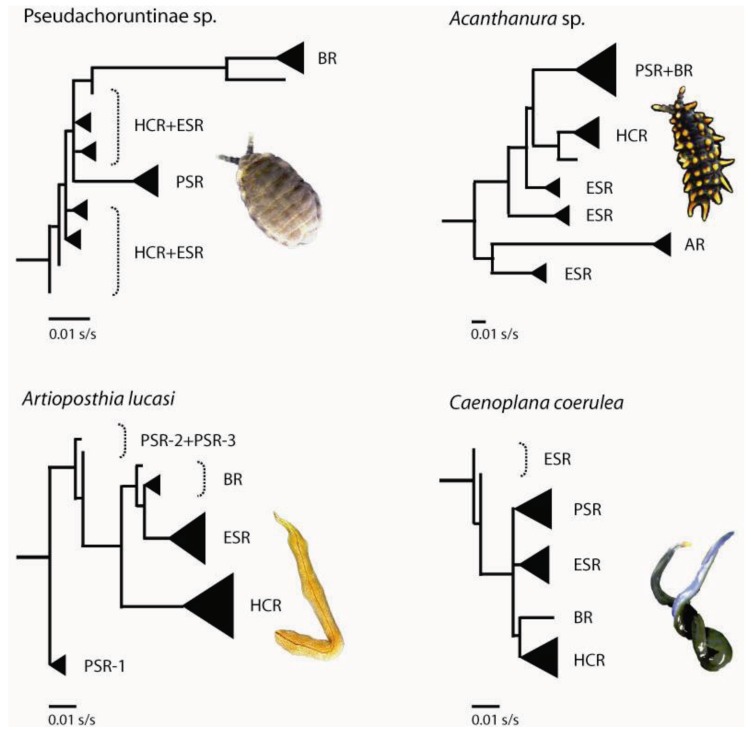
Simplified mtDNA gene trees that show evolutionary relationships among lineages from four co-distributed invertebrates. Phylogenies are out-group rooted, and were estimated using maximum likelihood, with the best-fit substitution model [74,76,77]. Solid black triangles represent sets of haplotypes that form monophyletic clades. Branch lengths are shown as substitutions per site (s/s). Population abbreviations follow Figure 3.

*Springtails*: Pseudachorutinae sp. is composed of four genetic clusters based on nuclear genotypic data (6 loci [74]), each broadly corresponding to a single *a priori* microgeographic region (*i.e.*, HCR, ESR, PSR and BR; Figure 1 and Figure 3). Anembo Region on the western side of the ridgeline, however, did not harbour a genetically distinct lineage. Also, and in contrast to our prior expectations, the Eastern Slopes Region did not contain the highest diversity of mtDNA sequences. Instead, based on the number of different haplotypes present, as well as the level of sequence divergence among them (as measured by H_d_ and *p*-dist, respectively), the Harolds Cross and Badja Regions are the most genetically diverse (Table 1). Furthermore, haplotypes sampled from Badja Region individuals are most phylogenetically distinct, separated from all others by a long branch—a pattern supported by nuclear DNA sequences [60,74,75]. While some Eastern Slopes (and Harolds Cross Region) haplotypes are early-branching in the outgroup-rooted mtDNA tree [74] (Figure 4), their phylogenetic position remains tentative given relatively low bootstrap support for this topology (not shown).

**Table 1:** Within-population diversity of mtDNA sequences. Names of genetic populations follow our previous publications on springtails [60,74,76] and flatworms [77], and generally correspond with *a priori* regions (see Figure 1). *N* is the sample size, *N*_hap_ is the number of different haplotypes detected in each population sample; H_d_ is haplotypic diversity (*i.e.*, the probability that two randomly selected sequences are different), and *p*-dist. is the mean sequence divergence among haplotypes with genetic distances measured as proportion of nucleotide differences and uncorrected for multiple substitutions at a single position in the alignment. Summary statistics could not be calculated for some populations (‘–’) because there was no genetic variation or too few sampled individuals. Analysis methods are described in online Supplementary Material.

	Pseudachorutinae sp.			*Acanthanura* sp.			Artioposthia lucasi*			*Caenoplana coerulea*		
Genetic population	*N* (*N*_hap_)	H_d_	*p*-dist.	*N* (*N*_hap_)	H_d_	*p*-dist.	*N* (*N*_hap_)	H_d_	*p*-dist.	*N* (*N*_hap_)	H_d_	*p*-dist.
Harolds Cross Region	81 (12)	0.795	0.003	25 (11)	0.880	0.013	64 (6)	0.379	0.006	80 (3)	0.251	0.001
Eastern Slopes Region	61 (7)	0.538	0.001	19 (7)	0.819	0.052	105 (5)	0.527	0.006	47 (5)	0.375	0.001
Pikes Saddle Region	112 (11)	0.280	0.004	66 (25)	0.891	0.023	21 (2)	0.095	0.001	47 (4)	0.537	0.002
Badja Region	64 (9)	0.792	0.009	78 (21)	0.903	0.010	–	–	–	–	–	–

* Summary statistics for the *A. lucasi* Pikes Saddle Region genetic population relate to PSR-1 in Fig. 3

*Acanthanura* sp. is divided into five distinct genetic clusters (four based on nuclear genotypic data alone, with a fifth cluster—Harold’s Cross Region—distinguished on the basis of a monophyletic mtDNA clade [75,76]). The geographic location of each largely corresponds with a different *a priori* catchment-based region (*i.e.*, HCR, ESR, AR, PSR and BR; Figure 1 and Figure 3). While the *Acanthanura* sp. genetic groups appear to be spatially congruent with those seen in Pseudachorutinae sp. (with the exception of Anembo Region), boundary overlap analyses revealed slight but significant differences in the spatial locations of genetic breaks [74]. Sequence divergence among mtDNA haplotypes is highest in Eastern Slopes Region—the predicted major refuge (Table 1). This was also the only a *priori* region found to contain haplotypes from more than one major mtDNA clade. In particular, *Acanthanura* sp. has three well-supported major clades in the Eastern Slopes Region, and all are relatively early-branching in the outgroup-rooted tree [76] (Figure 4). Surprisingly, mtDNA sequences from the Anembo Region *Acanthanura* sp. are also early-branching and clearly the most phylogenetically distinct, suggesting that this lineage is of considerable age under neutral assumptions.

*Flatworms.* Mitochondrial DNA sequences from *Artioposthia lucasi* reveal deep phylogeographic structure, with most sequences forming clades with non-overlapping spatial distributions, each restricted to Harolds Cross, Eastern Slopes or Badja Region [77] (Figure 3 and Figure 4). Some substructure is evident within Pikes Saddle; this region harbours a set of early-branching mtDNA haplotypes (note that this diversity is not evident in Table 1 because only the ‘PSR-1’ individuals were analysed using summary statistics). The spatial extent of the *Artioposthia lucasi* Eastern Slopes genetic group is similar to that of the *a priori* landscape model for Tallaganda, and also as expected, haplotypic diversity was quite high (Table 1; Figure 3). However, the Badja Region genetic group appears much more narrowly distributed than was predicted, and although poorly defined, the location of the Pikes Saddle / Badja Region genetic breaks seems to be further south than in other species examined to date. The more mobile flatworm, *Caenoplana coerulea*, also shows some phylogeographic structuring at Tallaganda, albeit with comparatively shallow divergences among mtDNA groups [77] (Table 1, Figure 4). Four genetic groups can be recognised (HCR, ESR, PSR and BR; Figure 1 and Figure 3). Interestingly, two of these groups—Harolds Cross and Eastern Slopes Region gene pools—have partly overlapping (parapatric) spatial distributions approximately mid-way along the north-south axis of Tallaganda. This pattern seems atypical of other taxa, although denser sampling of individuals may be required to confirm strictly allopatric distributions of genetic lineages detected in other species. Although there were few DNA sequence mutations distinguishing different haplotypes within a given region (Table 1), the Pikes Saddle Region (and to a lesser extent, Eastern Slopes) population had relatively high haplotypic diversity. The spatial location of the *Caenoplana coerulea* Pikes Saddle / Badja Region genetic break was again very similar to that seen in each of the two springtails, and also a co-distributed water skink [52] (Figure 3).

## 4. Past Events that Shaped Spatial-genetic Patterns

### 4.1. Long-term Persistence at Tallaganda

Notwithstanding challenges in estimating absolute divergence times from molecular data [78,79,80], DNA sequence datasets for the two springtail species indicate that population divergences are of considerable age. For example, substitution rates at least an order of magnitude faster than Brower’s [81] standard mtDNA rate estimate of 2.3% per million years must be invoked to reconcile observed net sequence divergences among major clades of *Acanthanura* sp. with an origin of lineage splitting as recent as mid-Pleistocene periglaciation events (*c*. 162 thousand years before present, recorded from the Tasmanian highlands [82]). Indeed, there is some support for a Late Pliocene / Early Pleistocene coalescence of mtDNA haplotypes in both springtail species [60,76]. Furthermore, similar indications of long-term occupancy have been inferred from mtDNA sequences of the two terrestrial flatworms. If we tentatively assume the substitution rate of Brower [81] as above, lineage divergence may date back to the late Pliocene for *Artioposthia lucasi*, and the mid-Pleistocene for *Caenoplana coerulea* [77]. Although springtails and terrestrial flatworms show different overall durations of occupancy at Tallaganda, they nonetheless have a long history of co-association and have persisted *in situ* through several (perhaps many) glacial-interglacial cycles of the Pleistocene.

### 4.2. Repeated Population Isolation and Expansion

There is strong evidence for multiple, recurrent impacts of Pleistocene climatic cycles on phylogeographic structure of the two springtail species [60,74,76], and similar conclusions were reached for co-distributed flatworms [77]. Phylogeographic inferences are consistent with alternation between fragmentation and range expansion events that most likely correspond with periods of climate-induced contraction and isolation of moist forest habitats during cool, dry periglacials, followed by expansion out of refugia during interglacials when forest connectivity was maximal [36]. Taken together, this indicates that at least some of Tallaganda’s catchment-based microgeographic regions were not completely deforested during the ~19 glacial-interglacial cycles of the Pleistocene [59]. The notion that at least some forest fragments persisted during periglacial climatic extremes in the south-eastern Australian highlands is supported by recent phylogeographic studies conducted over larger geographic scales in the region [54,55].

### 4.3. Number and Locations of Moist Forest Refuges at Tallaganda

Set in the context of topographically heterogeneous, low- to moderate-elevation landscapes on the Great Dividing Range, Tallaganda seems to have harboured several moist forest refuges that supported viable populations of saproxylic invertebrates—refuges that were most likely retained in sheltered gullies [36,57]. As predicted on the basis of topography and an assessment of the likely palaeoclimatic history of Tallaganda, the Eastern Slopes Region contained at least one high-quality refuge for both springtail species (probably three for *Acanthanura* sp. [76]). Indeed, this region appears to have acted as the major source of migrants into neighbouring catchments for Pseudachorutinae sp., at least over relatively short to intermediate evolutionary timescales [74]. There is also evidence that the Eastern Slopes Region served as an important refuge for terrestrial flatworms [77], suggesting that the *a priori* palaeoclimatic landscape model for Tallaganda may have broad utility for predicting patterns of local endemism in other wet-adapted saproxylic invertebrates.

Badja Region is particularly notable both in terms of its uniqueness, and the abrupt transition between it and the more northern genetic populations seen in several of the taxa studied. This microgeographic region was predicted to have been treeless steppe during periglacial activity owing to its low elevation [60], and given its connectivity with the Great Dividing Range to the south, recolonisation from *ex situ* sources cannot be ruled out. However, our phylogeographic inferences have provided some indications of within-region refugia. For example, relatively deep sequence divergences among Badja Region mtDNA haplotypes seen in Pseudachorutinae sp. [60,74], *Artioposthia lucasi* [77], the funnel web spider *Atraxsutherlandi* [83,84], and considerable mitochondrial and nuclear genetic diversity in *Acanthanura *sp. [76], are inconsistent with expectations for a recent recolonisation [45,47,48]. Furthermore, inferences of successive *Acanthanura* sp. range expansion events originating within Badja Region [76] indicate the retention of at least some moist forest habitats in that region. In contrast, *ex situ* sources of genetic diversity seems particularly plausible for the highland water skink *Eulamprus tympanum*, which shows a marked phylogeographic break at the same geographic location as the aforementioned invertebrates, but it is not restricted to Tallaganda and has sufficient dispersal capabilities to permit relatively rapid recolonisation from moderate- to high-elevation southern refuges [52]. Similarly, a recent recolonisation of Badja Region, originating from the Great Dividing Range outside the immediate Tallaganda system, is a plausible interpretation of the data for the flatworm *Caenoplana coerulea *[77].

Phylogeographic data indicate that Harolds Cross and Pikes Saddle Regions retained pockets of moist forest in the Pleistocene that acted as sources for local recolonisation following climatic amelioration [76,77]. In the case of *Acanthanura* sp., the Pikes Saddle refuge(s) may have been particularly favourable over recent periods, as indicated by this region’s inferred role as a major source of migrants into the neighbouring Eastern Slopes Region [74]. Although the comparatively dry and open-canopied Anembo Region on the western face of Tallaganda was not expected to contain any suitable Pleistocene forest refuges, it may have provided sufficient habitat to prevent local extinction of *Acanthanura* sp., of which it harbours a highly distinctive lineage [76]. Morphological and nuclear genetic distinctiveness of the velvet-worm *Euperipatoides rowelli* from Anembo Region [85,86] also supports the notion that saproxylic invertebrates persisted there.

### 4.4. Pseudo-Congruence and Incongruence

We have highlighted cases of congruence in spatial patterns of genetic diversity seen among co-distributed invertebrates at Tallaganda (Section 3.1) and apparent similarities in the underlying processes (Section 4.2) and the locations of refuge areas that generated and maintained biodiversity (Section 4.3). However, the extent to which the shared landscape setting, coupled with a reliance on dead wood, promoted concerted evolutionary responses among different members of the same ecological community deserves further consideration.

Indications that similar spatial-genetic patterns may have emerged via different underlying processes come from a qualitative comparison of the topologies of outgroup-rooted mtDNA gene trees (Figure 4). For example, both of the springtail species have gene trees where southern Badja Region haplotypes form late-branching clades, whereas the flatworm species have northern (*i.e.*, Harolds Cross Region) haplotypes as late branching. If we assume that phylogenetic sister lineages are likely to be geographic neighbours (*i.e.*, repeated long-distance range shifts of diverging clades are rare), then these contrasting patterns may indicate different geographic origins and spatial progressions of lineage emergence along the north-south axis of Tallaganda. There are also differences between members of the same taxon pair. In the springtails, *Acanthanura* sp. haplotypes from Pikes Saddle and Badja Regions are nested together in a well-supported monophyletic clade, yet no such higher-level relationship can be seen among Pseudachorutinae sp. haplotypes sampled from those same regions, based on the maximum likelihood estimate of the gene tree. That said, the latter phylogeny contains many short internodes, making the tree somewhat unstable, to the extent that topological congruence with the *Acanthanura* sp. tree cannot be rejected [74].

**Figure 5:**
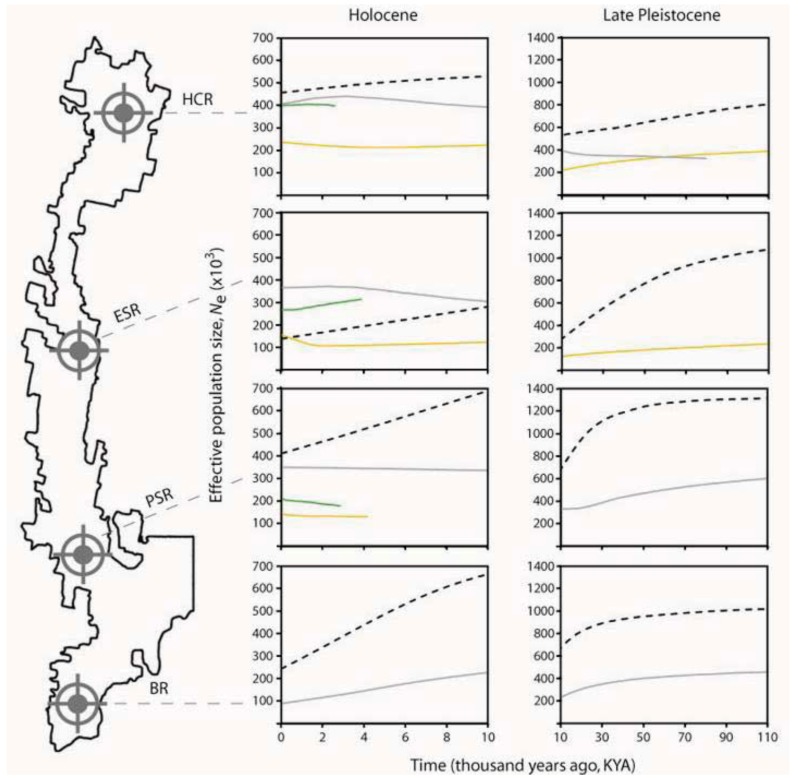
Bayesian Skyline plots showing changes in effective population size (*N*_e_), estimated from mtDNA sequences of co-distributed springtails and flatworms. Genetic population abbreviations follow Figure 3. Curves represent the median *N*_e_-value (*y*-axis) plotted over time (*x*-axis). Curve colours approximate colours of the species themselves (see Figure 2) and are as follows: Pseudachorutinae sp., pale gray; *Acanthanura* sp., black (dashed); *Artioposthia lucasi*, yellow; and *Caenoplana coerulea*, dark green (*A. lucasi* population PSR corresponds with PSR-1 in Figure 3). Note that for a given population, Holocene *vs*. Late Pleistocene represents the same analysis (rescaled). Associated confidence intervals are provided in online Supplementary Material (Figure S1). Some demographic reconstructions show very limited temporal depth given that there were few haplotypes (*N*_hap_) and the mtDNA sequence divergence among them was low (see Table 1). Bayesian Skyline plots could not be estimated for the two flatworm species’ Badja Region populations because there was no genetic variation. Analysis methods are described in online Supplementary Material.

Inferences about the nature and magnitude of changes in effective population size (*N*_e_) over time for springtails and flatworms reveal subtly different demographic histories among co-occurring populations (Figure 5). For example, throughout the Late Pleistocene, Harolds Cross Region populations of *Acanthanura* sp. (black, dashed) and *Artioposthia lucasi* (yellow) show parallel moderate declines, whereas Pseudachorutinae sp. (pale gray) seems to have been relatively constant (Figure 5, top right panel). Interestingly, despite quite disparate *N_e_* point estimates at earlier times, the two springtails converge towards very similar effective sizes during the Holocene warming (*i.e.*, last 10 thousand years, KY; Figure 5, top left panel). Notable contrasts are also seen in other regions of Tallaganda. In Eastern Slopes Region, *Acanthanura* sp. shows a threefold reduction in *N_e_* over the past ~100 KYA whereas *Artioposthia lucasi* remained quite stable. As above, despite different deep-time demographic histories, both of these species attained essentially the same *N_e_* in more recent times (Figure 5, second down, left panel). Deep-time contrasts and shallow-time convergences are also evident in springtails from Pikes Saddle and Badja Regions. Thus, together with among-taxon differences in the topologies of estimated mtDNA gene trees (Figure 4), these historical demographic analyses lend support to the idea that different underlying histories can in some cases yield superficially similar patterns of genetic diversity (or spatial structuring). However, here these Bayesian Skyline plots are intended only as a coarse, qualitative approach to comparing past demography, and we caution against over-interpretation of apparent trends. Most notably, all analytical methods used to investigate changes in *N,sub>e* over time assume that the basic underlying unit is a set of individuals randomly sampled from a panmictic population. Even relatively subtle substructure may bias estimates of model parameters, and so differences in within-region levels of substructure would need to be adequately accounted for prior to formal comparison. Since this cannot be achieved using the present genetic datasets, these Skyline plots are best viewed as hypothesis-generating, with inferences to be re-tested. 

Aside from cases of possible pseudo-congruence seen in springtails and flatworms, there are also some clear examples of phylogeographic incongruence. Indeed, while there is evidence for long-term persistence of some taxa at Tallaganda (Section 4.1), this is not true of all species pairs examined so far. For example, the log-dwelling funnelweb *Hadronyche cerberea* shows surprisingly shallow mtDNA sequence divergence and lacks phylogeographic structure, indicating recent recolonisation of Tallaganda [83,84]. Another example comes from more mobile vertebrates [52]. Here, mtDNA sequences from the southern water skink *Eulamprus heatwolei* suggest a very short history of this species at Tallaganda, perhaps post-dating the Last Glacial Maximum, whereas the morphologically similar highland water skink *Eulamprus tympanum* is much more deeply structured. Overall, incidences of phylogeographic incongruence among taxon pairs probably stems from subtle differences in microhabitat preferences and/or dispersal ability [52,74,77].

## 5. Contemporary Gene Flow Dynamics that Maintain Genetic Contact Zones

**Figure 6:**
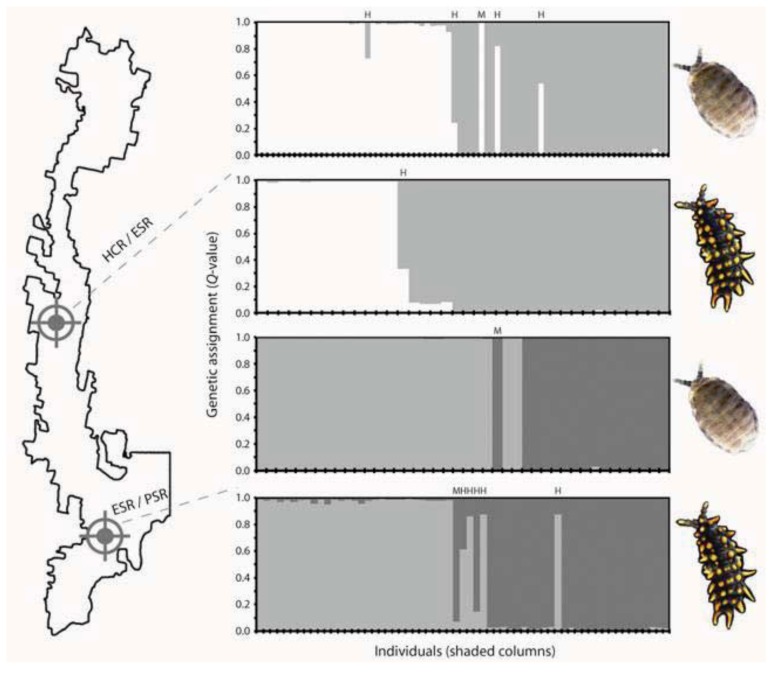
Gene flow dynamics across genetic contact zones within and among two co-distributed springtails. Left: Map showing approximate locations of spatially abrupt transitions between genetic populations (abbreviations follow Figure 3, and ‘/’ represents a break). Right: Bayesian clustering [93] of nuclear genotypic data (six loci per species) was used to perform genetic assignments of conspecific individuals. Plots show estimated membership coefficient (*Q*-value, *y*-axis) for each individual (columns, *x*-axis) collected from rotting logs at or near the location of a genetic contact zone, arranged sequentially from north to south. Columns are horizontally divided into two shaded segments, where area of the shading is proportional to the individual’s membership in each genetic population (white, Eastern Slopes Region; pale gray, Pikes Saddle Region; dark gray, Badja Region). Inferred migrant individuals (*i.e.*, those that have physically moved out of their population of origin and into the neighbouring population) are denoted by ‘M’, and inferred hybrids (either first-generation hybrids resulting from a cross between members of the two different genetic populations, or hybrids that result from subsequent backcrossing) are denoted by ‘H’. Analysis methods are described in online Supplementary Material.

The locations of genetic contact zones do not coincide with marked environmental gradients, and so it is possible that the relatively narrow, abrupt phylogeographic breaks detected in the two springtail species persist via some level of endogenous selection. Tension zones are maintained by the opposing forces of selection against hybrids and dispersal of individuals [87]. Thus, when multiple hybrid zones are present within a species, they do not necessarily exhibit the same dynamics: zones may differ in age and in width, depending on the interaction between selection intensity and dispersal capability [88,89]. Indeed, contrasting gene flow dynamics at different contact zones are apparent in both springtail species examined at Tallaganda (Figure 6). For example, nuclear genotypic data from Pseudachorutinae sp. reveal several hybrids and at least one migrant individual in the Eastern Slopes / Pikes Saddle contact zone samples, indicating rare on-going gene flow. Conversely, at the Pseudachorutinae sp. Pikes Saddle / Badja contact zone, there is no evidence for on-going gene flow, despite the presence of a migrant individual. The latter suggests that reproductive isolating mechanisms have developed in this part of the species’ range. The *Acanthanura* sp. Pikes Saddle / Badja contact zone is generally more diffuse, in the sense that at least one hybrid (probably a second generation backcross) was sampled within the geographic range of purebreds, rather than at the centre of the contact zone itself. Overall, contemporary migration and among-population gene flow in Pseudachorutinae sp. and *Acanthanura* sp. appears to be limited, such that effective migrants are probably too few to counteract the effects of genetic drift. The role of incomplete pre- or post-mating isolating mechanisms in shaping genetic structures of the two springtail species requires further investigation.

Reproductive isolating mechanisms that developed during long-term separation in montane forest refugia can preserve the signature of more ancient divergences following secondary contact. For example, Nalepa *et al*. [68] reported abrupt geographic transitions between different karyotypic races of the flightless saproxylic cockroach *Cryptocercus punctulatus* from the southern Appalachians—a biogeographic landscape with a qualitatively similar palaeoclimatic history and topographic complexity as Tallaganda. For this rotting-log-adapted invertebrate, the absence of contemporary physical or ecological barriers to dispersal that coincide with contact zones implicates partial or complete reproductive isolation [68]. While the origin and maintenance of well-defined hybrid zones is not always clear, such zones are not uncommon in terrestrial invertebrates. For example, Mesibov [90] identified a zone of contact approximately 100 metres wide, stretching for 230 km between two millipede species in Tasmania, Australia. Similarly, Shaw and Wilkinson [91] identified an extremely narrow hybrid zone (only 200 metres wide) between Moreton and Torresian chromosomal taxa of the grasshopper *Caledia captiva* in south-eastern Queensland, Australia. At that site, F2 progeny were found to be completely inviable owing to arrested development during embryogenesis, and the high level of mortality was ascribed to the production of novel imbalanced recombinant genotypes in the F1 generation. Endogenous selection has also been proposed to account for fine-scale contact zones among subspecies of the neanurid Collembolon *Monobella grassei* in the eastern Pyrenees [92], and at Tallaganda, the velvet-worm *Euperipatoides rowelli* shows severe developmental abnormalities at one well-characterised hybrid zone [86].

## 6. Conservation Considerations

### 6.1. The Role of Phylogeography in Protecting Saproxylic Invertebrate Biodiversity

A solid foundation of empirical research is central to mitigating negative anthropogenic impacts on genetic diversity [94]. Saproxylic invertebrates encompass a large proportion of biodiversity in Australian native forests [95,96,97], but because they may show variation and diversity over very fine spatial scales [69,98,99], conservation planning that focuses on the preservation of diversity of vertebrates and vascular plants is likely to be inadequate. Although the efficacy of landscape surrogates as ecological indicators (e.g., structural complexity, floristic composition, connectivity, living tree basal area, coarse woody debris (fallen timber) volume [43,100]) remains to be validated [101], landscape history may be particularly useful in the case of wet-adapted saproxylic invertebrates. For example, in the Australian Wet Tropics, Graham *et al*. [35] showed that rainforest stability throughout the Late Quaternary was the most important determinant of contemporary spatial patterns of biodiversity in low-mobility organisms (relative to other variables examined). Indeed, by explicitly considering landscape history, comparative phylogeographic approaches are well-suited to identifying areas that represent irreplaceable (vicariant) genetic diversity [4,15].

### 6.2. The Current Reserve System at Tallaganda

**Figure 7:**
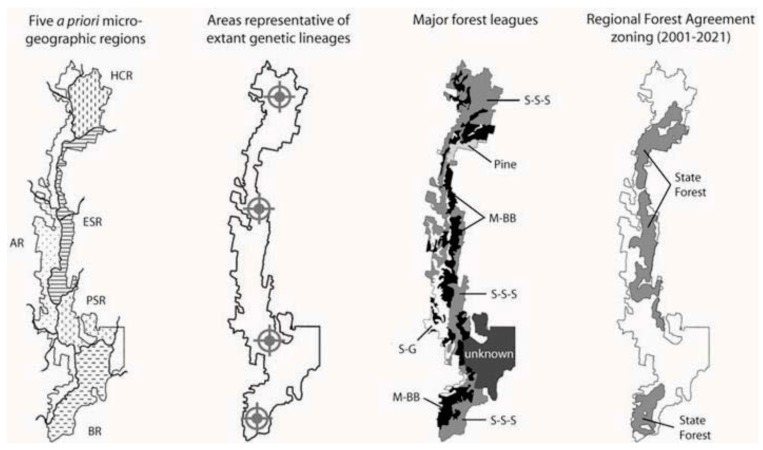
Comparison of several alternative landscape classification schemes with the Australian Government’s [103] Region Forest Agreement zoning of Tallaganda. Far left. Five *a priori* microgeographic regions are based largely on topography, as well as broad understanding of palaeoclimatic changes in forest distributions and connectivity (see section 1.4; Figure 1). Left of centre: The areas marked with target symbols and identified as being ‘*representative*’ of evolutionarily distinct lineages were determined from the spatial distributions of genetic populations in five species (Figure 2). Locations are approximate, and alternative configurations may capture similar amounts of intraspecific diversity. Right of centre: The distribution of major forest leagues simplified from State Forests of New South Wales [105]. Abbreviations are: Scribbly Gum-Stringybark-Silvertop Ash, S-S-S; Messmate-Brown Barrel, M-BB; Snowgum, SG; commercial pine plantation, Pine. Far right: Areas of Tallaganda zoned as State Forest under the current Regional Forest Agreement’s *‘comprehensive, adequate and representative*’ reserve system.

Long-term intensive timber harvesting in Europe has resulted in marked declines in forest invertebrate diversity, and a disproportionately large number of threatened or endangered insects are saproxylic [102]. Although production forestry in Australia has a short history, habitat fragmentation processes are already evident [43]. In New South Wales, the rubric of a ‘*comprehensive, adequate and representative*’ (CAR) reserve system was established as a framework for sustainable forest management. The Regional Forest Agreement for southern New South Wales [103], which will remain in force for the subsequent 20 years, resulted in the rezoning of several parts of Tallaganda (Figure 7). Effectively all of the Eastern Slopes Region—the area that this and related work has demonstrated to harbour the most highquality forest refuges for saproxylic invertebrates—was zoned as State Forest, and is therefore subject to intensive commercial forestry operations that will continue at least for the duration of the current Regional Forest Agreement. Under current practices of short (*i.e.*, 80 to 100-year) logging cycles, temporal continuity of large-diameter rotting logs cannot be assured [41,97,104]. The degree to which areas of Tallaganda that were zoned as Dedicated Reserves (*i.e.*, National Parks, Nature Reserves and Flora Reserves) are ‘*representative*’ of extant floristic biodiversity is also questionable, even at a coarse level of classification. For example, the Messmate-Brown Barrel forest league (high-quality, moist sclerophyll forest 40–50 metres tall) is poorly represented in the reserve system. Conversely, the Scribbly Gum-Stringybark-Silvertop Ash (mainly dry sclerophyll forest types), and the Snowgum league (a limited range of cold-tolerant species with 10–20 metre canopies) are proportionately overrepresented at Tallaganda (Figure 7). That said, the latter are important communities in their own right, and should be afforded some protection.

### 6.3. Ameliorating the Potential Negative Impacts of Commercial Forestry

Natural forest dynamics and ecological processes might be maintained in production forests if ‘low-impact’ management practices are adopted. Spatial connectivity of dead wood can be promoted by allowing fallen logs to decay *in situ*, by conducting clear felling on relatively small spatial scales, and by favouring harvesting methods that result in a mosaic of forest age classes (e.g., selective logging). Temporal continuity of large-diameter rotting log recruitment can be promoted by leaving some commercially over-mature trees standing, and using longer logging rotation cycles. Importantly, these measures need not compromise the ability of commercial forestry operations to generate an economic return on timber harvesting [106]. Indeed, Tallaganda has been logged for >150 years and still has a rich invertebrate biodiversity, although forestry practices have become increasingly intensive [103,105]. Adaptive management strategies that incorporate the best available information on the spatial distribution of biodiversity, species’ sensitivity to habitat fragmentation (e.g., dispersal abilities, migration rates, genetic neighbourhood sizes), and the locations of flora and fauna refuges, are essential for achieving an ecologically sustainable forestry industry [107,108].

## 7. Conclusions

The identification of centres of biodiversity in distantly-related, co-distributed members of the same ecological community has conservation applications, particularly for prioritisation of areas for protection. Species with characteristics such as low mobility, high ecological specialisation, and long-term occupation of a region, are well-suited for inferring local landscape history via the genetic signatures of past range expansion and contraction, population divergence, and gene flow. Saproxylic invertebrates may be exceptional in their ability to recover fine-scale signal given that viable populations may persist in small forest refuges—refuges too small to support most vertebrates during Pleistocene ice ages. At Tallaganda, marked fine-scale population sub-structuring, large intraspecific divergences, and strong spatial associations between ancient phylogeographic breaks plus contemporary population boundaries with *a priori* catchment-based microgeographic regions were detected. Partly concerted responses to Pleistocene glacial-interglacial cycles may be attributable to inherent features of species’ biology (e.g. poor dispersal ability) together with a common landscape experience, underpinned by a shared reliance on cool and moist rotting log microhabitats.

## Supplementary Files

Supplementary File 1
