# Supplementary material for: Phylogeography of Saproxylic and Forest Floor Invertebrates from Tallaganda, South-eastern Australia
Source: Insects. 2012 Feb 29;3(1):270–94. doi: 10.3390/insects3010270 (PMC4553628; doi:10.3390/insects3010270)
Supplement: Supplementary File 1 [file insects-03-00270-s001.pdf]

Insects **2012**, *3*, 1-5

OPEN ACCESS

insects

ISSN 2075-4450

www.mdpi.com/journal/insects/

Review

Phylogeography of Saproxylic and Forest Floor Invertebrates from Tallaganda, South-eastern Australia

Ryan C. Garrick [1,2,*], David M. Rowell [3] and Paul Sunnucks [4]

[1] Department of Ecology and Evolutionary Biology, Yale University, New Haven, CT 06520, USA
[2] Present address: Department of Biology, University of Mississippi, University, MS 38677, USA
[3] Department of Evolution, Ecology and Genetics, Research School of Biology, Australian National University, Canberra, ACT 0200, Australia; E-Mail: David.Rowell@anu.edu.au
[4] Australian Centre for Biodiversity, School of Biological Sciences, Monash University, Clayton, VIC 3800, Australia; E-Mail: paul.sunnucks@monash.edu

* Author to whom correspondence should be addressed; E-Mail: rgarrick@olemiss.edu

Received: 03 January 2012; in revised form: 21 February 2012 / Accepted: 22 February 2012 / Published: 29 February 2012

Abstract: The interaction between physiogeographic landscape context and certain life history characteristics, particularly dispersal ability, can generate predictable outcomes for how species responded to Pleistocene (and earlier) climatic changes. Furthermore, the extent to which impacts of past landscape-level changes 'scale-up' to whole communities has begun to be addressed via comparative phylogeographic analyses of co-distributed species. Here we present an overview of a body of research on flightless low-mobility forest invertebrates, focusing on two springtails and two terrestrial flatworms, from Tallaganda on the Great Dividing Range of south-eastern Australia. These species are distantly-related, and represent contrasting trophic levels (*i.e.*, slime-mold-grazers vs. higher-level predators). However, they share an association with the dead wood (saproxylic) habitat. Spatial patterns of intraspecific genetic diversity partly conform to topography-based divisions that circumscribe five 'microgeographic regions' at Tallaganda. In synthesizing population processes and past events that generated contemporary spatial patterns of genetic diversity in these forest floor invertebrates, we highlight cases of phylogeographic congruence, pseudo-congruence, and incongruence. Finally, we propose conservation-oriented recommendations for the prioritisation of areas for protection.

Keywords: biodiversity; dead wood; endemism; montane refuges; population genetics

Supplementary Material

1. Methods

1.1. Data Availability

As part of our review paper, we performed some re-analyses of published datasets. GenBank accession numbers for mitochondrial DNA (mtDNA) sequences from Collembola are given in Garrick *et al.* [1–3], and accessions for flatworm mtDNA sequences can be found in Sunnucks *et al.* [4]. Collembola nuclear genotypes used here in clustering analyses are available on request.

1.2. Genetic Diversity Summary Statistics

To quantify mtDNA sequence polymorphism within populations we used DnaSP v5.10 [5] to calculate the number different haplotypes (N_{hap}), haplotypic diversity (H_d; the probability that two randomly drawn sequences are identical), and the mean sequence divergence among haplotypes, as measured using uncorrected *p*-distances (*p*-dist.). The latter two summary statistics are standardized with respect to sample size, thereby facilitating direct comparison across populations and species.

1.3. Bayesian Skyline Plots

To reconstruct population size changes operating over long-term, evolutionary timescales, we used Bayesian skyline plots [6], implemented in BEAST v1.6.1 [7]. For these analyses, the basic underlying unit is assumed to be a single unstructured population. Putatively panmictic groups of springtails (Pseudachorutinae sp. and *Acanthanura* sp.) were inferred from genotypic clustering of nuclear genetic data. However, only mtDNA data were available for flatworms (*Artioposthia lucasi* and *Caenoplana coerulea*). In the latter cases, it was necessary to use phylogenetic lineages on an estimated gene tree (see Figure 4 of the main text) as basic unit of analysis. We recognize that neither of these approaches excludes the possibility of subtle within-group substructure, and thus results should be interpreted with caution. Searches used the GTR+I+G substitution model, empirical base frequencies, a strict-clock with Brower's [8] standard arthropod mtDNA rate ('clock.rate' = 1.15×10^{-8} with the simplifying assumptions of 1 year generation times for each species), a coalescent (Bayesian skyline) tree prior, linear skyline model, UPGMA-generated starting trees with 10 groups, 'skyline.popsize' with a uniform prior and bounded by 0 and 1.5×10^{6} (initial value = 10,000), auto-optimized tuning, and other priors as default. Final searches were performed using 1×10^{8} MCMC generations sampling parameters every 2,000th step (10% discarded as burn-in), and three replicate runs per dataset. One representative replicate run is presented per dataset, and confidence intervals around the median effective population size (N_e) value in these selected replicates are shown in Supplementary Figure S1.

Figure S1. Confidence intervals associated with Bayesian Skyline plots showing changes in N_e over time, based in mtDNA sequence data (Figure 5 of the main text). Curves represent the upper and lower 95% highest posterior density limits for N_e-values (*y*-axis) plotted over time (*x*-axis). Curve colours approximate colours of the species themselves (Figure 2 of the main text) and are as follows: Pseudachorutinae sp., pale gray; *Acanthanura* sp., black (dashed); *Artioposthia lucasi*, yellow; and *Caenoplana coerulea*, dark green (note that *A. lucasi* population PSR corresponds with PSR-1 in Figure 3 of the main text). Note that for a given population, Holocene *vs*. Late Pleistocene represents the same analysis (rescaled).

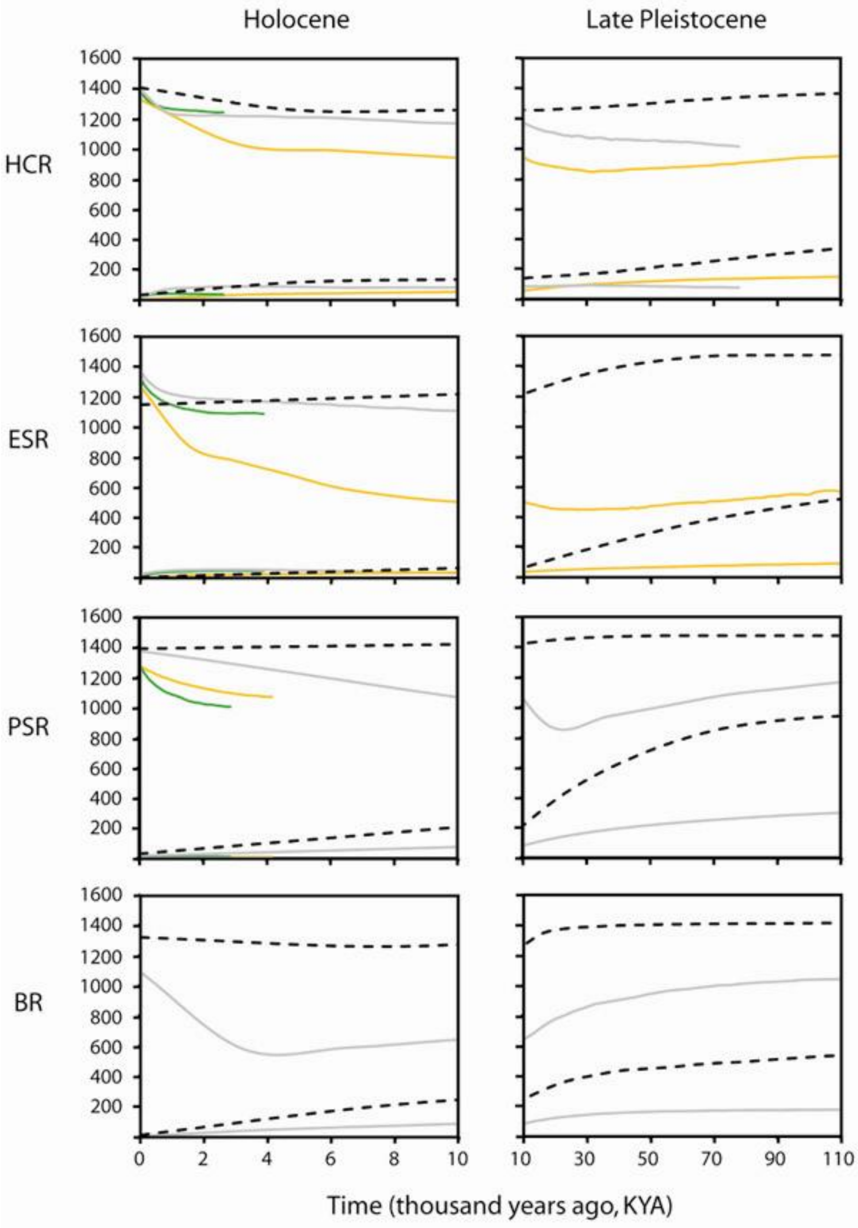

1.4. Contact Zone Dynamics

Here we focused on two previously identified locations at Tallaganda in which both springtail species show abrupt spatial transitions between genetic populations: the Eastern Slopes / Pikes Saddle

contact zone, and the Pikes Saddle / Badja contact zone [1–3]. For each species, we selected the subset of individuals that were collected at or near these zones. To reduce the impact of kin clustering on inferences about contact zone dynamics, in rotting logs for which ≥5 individuals were sampled, we used KINGROUP v2 [9] to identify putative full siblings. If detected, we retained one randomly selected representative of each family group. We then analysed these reduced datasets using STRUCTURE v2.2.3 [10] with the number of clusters (*K*) fixed at two. Searches used the 'correlated allele frequency' and 'admixture ancestry' models, 1×10^5 MCMC generations burn-in and a run length of 1×10^6 generations. STRUCTURE membership coefficients (*Q*-values) were used to classify an individual's ancestry as purebred ($Q \geq 0.9$), or hybrid ($Q < 0.9$), with hybrids being indicative of ongoing *gene flow* across the contact zone. Similarly, the presence of first-generation migrants—purebred individuals sampled at a spatial location that is characteristic of other genetic population's native range—is indicative of successful *dispersal* across the contact zone.

References

1. Garrick, R.C.; Sands, C.J.; Rowell, D.M.; Tait, N.N.; Greenslade, P.; Sunnucks, P. Phylogeography recapitulates topography: Very fine-scale local endemism of a saproxylic 'giant' springtail at Tallaganda in the Great Dividing Range of south-east Australia. *Mol. Ecol.* **2004**, *13*, 3329–3344.
2. Garrick, R.C.; Sands, C.J.; Rowell, D.M.; Hillis, D.M.; Sunnucks, P. Catchments catch all: Long-term population history of a giant springtail from the southeast Australian highlands—A multigene approach. *Mol. Ecol.* **2007**, *16*, 1865–1882.
3. Garrick, R.C.; Rowell, D.M.; Simmons, C.S.; Hillis, D.M.; Sunnucks, P. Fine-scale phylogeographic congruence despite demographic incongruence in two low-mobility saproxylic springtails. *Evolution* **2008**, *62*, 1103–1118.
4. Sunnucks, P.; Blacket, M.J.; Taylor, J.M.; Sands, C.J.; Ciavaglia, S.A.; Garrick, R.C.; Tait, N.N.; Rowell, D.M.; Pavlova, A. A tale of two flatties: Different responses of two terrestrial flatworms to past environmental climatic fluctuations at Tallaganda in montane southeastern Australia. *Mol. Ecol.* **2006**, *15*, 4513–4531.
5. Librado, P.; Rozas, J. DnaSP v5: A software for comprehensive analysis of DNA polymorphism data. *Bioinformatics* **2009**, *25*, 1451–1452.
6. Drummond, A.J.; Rambaut, A.; Shapiro, B.; Pybus, O.G. Bayesian coalescent inference of past population dynamics from molecular sequences. *Mol. Biol. Evol.* **2005**, *22*, 1185–1192.
7. Drummond, A.J.; Rambaut, A. BEAST: Bayesian evolutionary analysis by sampling trees. *BMC Evol. Biol.* **2007**, *7*,214.
8. Brower, A.V.Z. Rapid morphological radiation and convergence among races of the butterfly *Heliconius erato* inferred from patterns of mitochondrial DNA evolution. *Proc. Natl. Acad. Sci. USA* **1994**, *91*, 6491–6495.
9. Konovalov, D.A.; Manning, C.; Henshaw, M.T. KINGROUP: A program for pedigree relationship reconstruction and kin group assignments using genetic markers. *Mol. Ecol. Notes* **2004**, *4*, 779–782.

10. Pritchard, J.K.; Stevens, M.; Donnelly, P. Inference of population structure using multilocus genotype data. *Genetics* **2000**, *155*, 945–959.

© 2012 by the authors; licensee MDPI, Basel, Switzerland. This article is an open access article distributed under the terms and conditions of the Creative Commons Attribution license (http://creativecommons.org/licenses/by/3.0/).
